# Transforming Growth Factor-Beta Inhibition Reduces Progression of Early Choroidal Neovascularization Lesions in Rats: P17 and P144 Peptides

**DOI:** 10.1371/journal.pone.0065434

**Published:** 2013-05-31

**Authors:** Javier Zarranz-Ventura, Patricia Fernández-Robredo, Sergio Recalde, Angel Salinas-Alamán, Francisco Borrás-Cuesta, Javier Dotor, Alfredo García-Layana

**Affiliations:** 1 Experimental Ophthalmology Laboratory, Universidad de Navarra, Pamplona, Spain; 2 Department of Ophthalmology, Clínica Universidad de Navarra, Universidad de Navarra, Pamplona, Spain; 3 Division of Hepatology and Gene Therapy, Center for Applied Medical Research (CIMA), Universidad de Navarra, Pamplona, Spain; 4 Digna Biotech, Madrid, Spain; Massachusetts Eye & Ear Infirmary, Harvard Medical School, United States of America

## Abstract

The purpose of this study was to assess the effects of transforming growth factor beta (TGF-β) inhibitor peptides (P17 & P144) on early laser-induced choroidal neovascularization (LI-CNV) lesions in rats, two weeks after laser CNV induction. Seventy-one Long Evans rats underwent diode laser application in an established LI-CNV model. Baseline fluorescein angiography (FA) was performed 14 days following laser procedure, and treatments were administered 16 days post-laser application via different administration routes. Intravenous groups included control (IV-Control), P17 (IV-17), and P144 (IV-144) groups, whereas intravitreal groups included P17 (IVT-17), P144 (IVT-144), and a mixture of both peptides (IVT-17+144) (with fellow eyes receiving vehicle alone). CNV evolution was assessed using FA performed weekly for four weeks after treatment. Following sacrifice, VEGF, TGF-β, COX-2, IGF-1, PAI-1, IL-6, MMP-2, MMP-9, and TNF-α gene expression was assessed using RT-PCR. VEGF and p-SMAD2 protein levels were also assessed by western-blot, while MMP-2 activity was assessed with gelatin zymography. Regarding the FA analysis, the mean CNV area was lower from the 3^rd^ week in IVT-17 and IVT-144 groups, and also from the 2^nd^ week in IVT-17+144. Biochemical analysis revealed that gene expression was lower for VEGF and COX-2 genes in IV-17 and IV-144 groups, VEGF gene in IVT-17+144 group and MMP-2 gene in IVT-17 and IVT-144 groups. VEGF protein expression was also decreased in IV-17, IV-144, IVT-17 and IVT-144, whereas pSMAD-2 levels were lower in IV-17, IV-144 and IVT-17+144 groups. Zymogram analysis revealed decreased MMP-2 activity in IV-17, IV-144, IVT-17 and IVT-144 groups. These data suggest that the use of TGF-β inhibitor peptides (P17 & P144) decrease the development of early CNV lesions by targeting different mediators than those typically affected using current anti-angiogenic therapies. Its potential role in the treatment of early CNV appears promising as a single therapy or adjuvant to anti-VEGF drugs.

## Introduction

Choroidal neovascularization (CNV) is the final common process for multiple retinal diseases in which the development of abnormal neovascular tissue with increased permeability produces fluid leakage into the outer subretinal space in the macula [1]–[5]. In naïve lesions that remain untreated, CNV development is followed by fibrous processes that end in progressive macular destruction, leading to substantial loss of autonomy and quality of life [6], [7]. This multifactorial process involves multiple molecules in complex networks, the study of which has become a major area of research in recent years. In normal eyes, the retinal pigment epithelium (RPE) and other cells maintain a global antiangiogenic state in the resting retina. In CNV, upregulation of proangiogenic molecules such as vascular endothelial growth factor (VEGF) (the main factor related with angiogenesis), platelet-derived growth factor (PDGF), angiopoietin, and b-fibroblast growth factor (b-FGF), and decreased production of angiogenic inhibitors, such as pigment epithelium-derived factor (PEDF) and thrombospondin-1, produces an imbalance that favours neovascular growth [8]–[10]. The overexpression of VEGF in CNV has been demonstrated and this molecule has become the target of current therapies [11]–[14]. Recent studies have been directed towards other mediators related with VEGF-upregulation, among which is transforming growth factor beta (TGF-β)[15], [16]. TGF-β is a pleiotropic molecule that participates in both angiogenesis and fibrotic processes, and its presence has been demonstrated in surgically excised human neovascular membranes [17]. More recently, studies using human RPE cell cultures have reported that TGF-β significantly enhances VEGF secretion and also exerts a strong effect through extracellular matrix remodelling [15], [16], [18]. A synergistic association with b-FGF and tumor necrosis factor-alpha (TNF-α) over VEGF transcription has been reported, suggesting that TGF-β may work in concert with other cytokines in VEGF upregulation [19]. These findings led us to speculate that selective blockade of TGF-β would abate CNV development, a hypothesis recently confirmed in a study performed with two anti-TGF-β inhibitor peptides in a laser induced CNV model [20]. In this study, we evaluated the effect of these peptides on CNV induction, with treatments administered 48 hours after laser application. However, CNV development occurs in different stages, each of which is mediated by different growth factors and cellular types. Therefore, separate consideration of each stage should be considered for the development of optimal treatment strategies. Whereas some cytokines participate in several stages of CNV formation, as well as in a number of disparate but simultaneous processes, these molecules appear as the ideal target for CNV therapies. The multiple roles played by TGF-β suggest that its blockage may not only decrease the angiogenic process related with CNV, but may also affect other pathways related to progression of the disease.

To test this hypothesis, in this new study design we assessed the effect of the anti-TGF-β peptides in early CNV lesions 16 days after laser induction, in contrast to our previous study in CNV induction in which treatments were administered 48 hours post laser [20]. Whereas mostly of the studies in the literature have evaluated therapies administered around the time of lesion induction, recently a different behaviour of some treatments in early lesions at a later stage has been reported [21]. In the current work, the 16 days period of time after laser induction corresponds with the peak of CNV formation previously reported in our established laser-induced CNV animal model [22] and aims to reproduce early lesions similar to those seen in clinical practice.

We selected the two specific TGF-β inhibitor peptides employed in our previous study - P17 and P144 - at their most effective doses and we also include a recently developed intravenous solution of P144. Administration of these peptides is safe and has demonstrated a strong TGF-β inhibitory effect in different animal models, cellular cultures and *in vivo* angiogenesis assays in a synthetic matrix [23]–[28]. P17 inhibits TGF-β with a relative binding affinity of 100% for TGF-β_1_, 80% for TGF-β_2_ and 30% for TGF-β_3_ in surface plasmon resonance (SPR) assays [29], whereas similar binding assays are being currently carried out for P144 (unpublished data).

## Materials and Methods

### Study Animals

The current study was conducted according to the Association for Research in Vision and Ophthalmology (ARVO) Resolution on the Use of Animals in Ophthalmic and Vision Research and approved by the Ethics Committee for Animal Research of the University of Navarra. Seventy-one pigmented-retina Long Evans male rats (age, 12 weeks; weight, approximately 320 grams) were randomized in three intravenous (IV-Control, IV-17, IV-144) and three intravitreal (IVT-17, IVT-144, IVT-17+144) study groups. Rats were intraperitoneally anesthetized with a mixture of ketamine (Imalgene 1000) (75 mg/kg) and Xylacine (Rompun 2%) (10 mg/kg) for all procedures. Injections were performed with a 1 mL syringe and a 25-gauge needle.

### Diode Laser-Induced CNV Model

All study eyes were dilated with tropicamide 1% eye drops and eight to ten laser photocoagulation sites were placed concentrically around the optic disc for induction of CNV lesions. A diode laser (810 nm) connected to a slit lamp was used with a relative potency scale of 250 mW, an exposure time of 0.05 seconds, and a spot size of 75 µm as established by protocol [22]. Laser spots were focused with crystal covers to avoid laser beam dispersion. Bubble formation confirmed the rupture of Bruchs membrane. Laser rupture sites which developed haemorrhage or subretinal bleeding at the time of laser application were excluded from analysis. The mean leakage area of CNV lesions that developed at laser photocoagulation sites was then assessed at each time point. Differences between treated and control eyes for each study group were then analyzed.

### Study Groups

#### Intravenous Treatment Groups

Three intravenous groups were included in the study, control, P17 and P144 groups. The intravenous control group (IV-Control) received five intravenous injections of 0.2 mL of saline every 72 hours over 2 weeks, representative of natural laser-induced CNV evolution. Both intravenous P17 and P144 groups (IV-17 and IV-144 respectively) received five intravenous injections of 0.2 ml of P17 (1 mg/ml) or P144 (1 mg/ml) suspended in 0.2 mL of saline (P17) or carbonate (P144), every 72 hours over 2 weeks. Nine rats were included in the IV-Control group, 10 rats were included in the IV-17 group, and 10 rats were included in the IV-144 group.

#### Intravitreal Treatment Groups

Three groups were included; P17, P144 and a mixture of both peptides P17+P144. The dose and concentration of each peptide intravitreally injected was as follows: P17 (IVT-17; 20 mg/ml), P144 (IVT-144; 3 mg/ml) and P17+P144 (IVT-17+144; 20 & 3 mg/ml respectively). These rats received a single dose of 7 µl of peptide solution in the treated eye and 7 µl of its respective vehicle in the fellow eye of the same animal. Whereas the hydrophilic P17 peptide was suspended in saline, the hydrophobic P144 peptide and the mixture of both peptides were suspended in a 75% of dimethyl sulfoxide saline solution. Fourteen rats in IVT-17, 12 rats in IVT-144 and 16 rats in IVT-17+144 were included in the study groups.

### Injection Procedures

Treatments were administered 16 days after laser application in all study groups. Intravenous injections were performed with 1 mL syringes and 30G needle in the tail vein. Intravitreal injections were performed with high-precision Hamilton syringes (Gastight 1702LT Hamilton Co., Reno, NV) and 30 gauge needles. Injection sites were located 1 mm posterior to the corneoscleral limbus. A single 7 µl injection of different peptide concentrations was administered in the treated eye and the same volume of vehicle solution in the fellow eye of the same animal (intravitreal controls) in all intravitreal study groups (IVT-17, IVT-144, IVT-17+144). The treatment eye was randomly selected for each rat.

### Peptides

P144 (TSLDASIIWAMMQN), a hydrophobic peptide derived from the sequence of the extracellular region of type III receptor for TGF-β (Betaglycan, amino acids 730–743) [24], and P17 (KRIWFIPRSSWYERA), a soluble hydrophilic peptide identified using a random phage display peptide library [25], were synthesized by the solid phase method using the Fmoc alternative by PolyPeptide Laboratories (Strasbourg, France)[30], [31]. Peptides were at least 95% pure as per HPLC.

### Fluorescein Angiography (FA)

**Figure 1:**
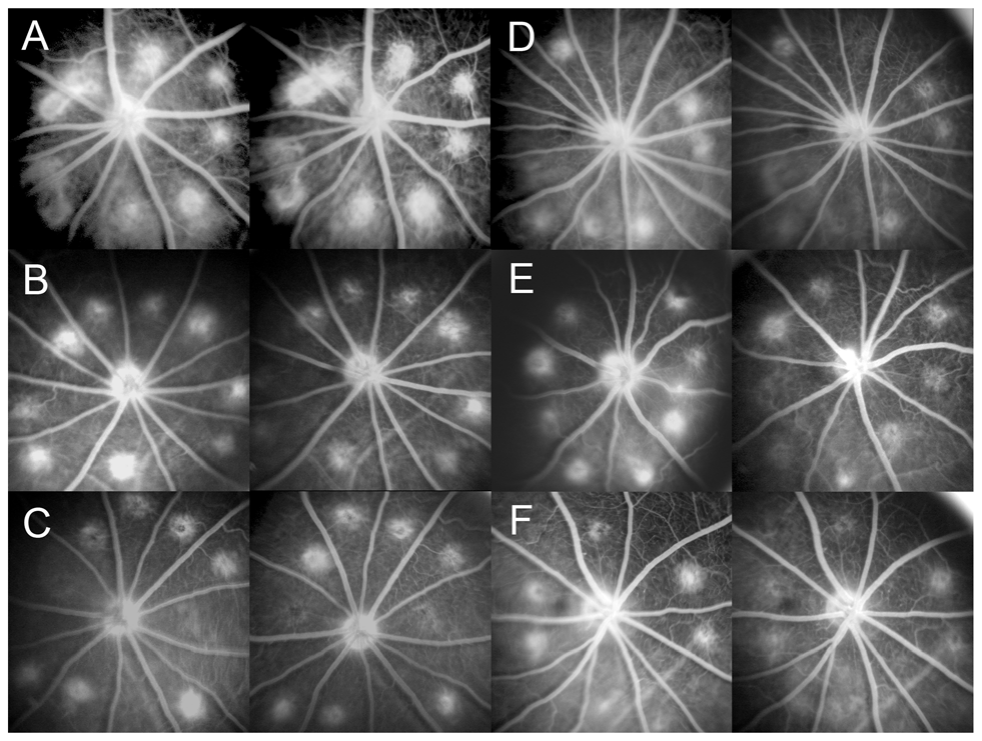
Fluorescein angiography (FA) images of all study groups. Left-image: Baseline, right-image: 4 weeks post-treatment. A: Intravenous control group (IV-Control). B: Intravenous P17 group (IV-17). C: Intravenous P144 group (IV-144). D: Intravitreal P17 group (IVT-17). E: Intravitreal P144 group (IVT-144). F: Intravitreal P17+P144 group (IVT-17+144).

Images were obtained using a digital-adapted Retinograph (Topcon TRC-50FX, Topcon Inc., Tokyo, Japan). For FA evaluations, 0.2 mL of intravenous sodium fluorescein 2% (200 mL/kg) was administered into the tail vein 2 minutes before the images were captured (Figure 1). Digital FA images were converted to TIFF files with Adobe Photoshop® 7.0 software (Adobe Inc., San Jose, CA, USA), and CNV leakage area was calculated measuring the pixel area within the best-fitting polygon of each rupture site [32] using the “shape definition” tool of ImageQuantTL® (Amersham Biosciences, Uppsala, Sweden) software by two independent, trained, masked observers (JZV, SR). Mean CNV area was determined for every study group and timepoint. FA was performed 2 weeks after laser induction (baseline) and weekly post-treatment injection for 4 consecutive weeks to assess the CNV evolution in all study groups.

### Quantitative Real time-Polymerase Chain Reaction (RT-PCR)

Total RNA was isolated from rat retina using ABI PRISM™ 6100 Nucleic Acid PrepStation (Life technology, Carlsbad, California, USA). 1000 ng of each mRNA was reverse transcribed with the qScript cDNA Supermix kit (Quanta Biosciences Inc., Gaithersburg, MD, USA) using *2720* Thermal cycler (Life technology, Carlsbad, California, USA). Nine pre-designed and validated gene-specific TaqMan Gene Expression Assays from the inflammation and vascularization pathways (Interleukin (IL)-6, MMP-2, MMP-9, TNF-α, COX-2, VEGF, TGF-β, TNF-α and PAI-1) from Applied Biosystem were used for quantitative real-time RT-PCR in ABI Prism 7300 real-time PCR system (Applied Biosystems). For further statistical evaluations the level of applied housekeeping gene (ACTB) was used as endogenous control for data normalization. Relative quantification studies were made from collected data (threshold cycle numbers, referred as C_t_) with 7300 System SDS software 1.3 (Applied Biosystems). Relative quantity (RQ) of the gene specific mRNA was calculated by DataAssist v 2.0.

### Western blotting for vascular endothelial growth factor (VEGF) and phosphorylated SMAD-2

Fiveµg of RPE-choroid homogenates were mixed with Laemmli buffer (Bio-Rad), boiled for 5 min and separated on 10–12% SDS-PAGE gels and transferred to a nitrocellulose membrane. After blocking with 5% skimmed milk (w/v), 0.1% Tween-20 (w/v) in TBS (1 hour, RT) membranes were exposed to the primary antibody anti-VEGF (0.2 µg/µL, monoclonal anti-VEGF, Santa Cruz Biotechnology Inc., Santa Cruz, CA) at RT for 1 hour followed by incubation at RT for 1 hour with a horseradish peroxidase-conjugated goat anti-mouse antibody (sc2005; 0.4 µg/µL, Santa Cruz Biotechnology Inc.). Membranes were tested for anti-Smad2, phospho-specific (Chemicon-Millipore, Billerica, MA) overnight at 4°C (0.5 µg/ml) followed by incubation at RT for 2 hours with a horseradish peroxidase-conjugated goat anti-rabbit antibody (sc2054; 0.4 µg/µL, Santa Cruz Biotechnology Inc.). Signals were detected with an enhanced chemoluminescence (ECL) kit (ECL Western blotting detection kit, GE Healthcare, Fairfield, CT) and captured with ImageQuant 400 (GE Healthcare). The relative intensities of the immunoreactive bands were analyzed with ImageQuantTL software (GE Healthcare). The loading was verified by Ponceau S red and the same blot was stripped and reblotted with an anti-ß-actin monoclonal antibody (Sigma-Aldrich) to normalize the VEGF and pSMAD-2 levels.

### Gelatin zymography assay for matrix metalloproteinase-2 (MMP-2) activity

MMP-2 activity was quantified by gelatin zymography on RPE-choroid homogenates. Fiveµg of total protein from homogenate supernatants were mixed with non-reducing sample buffer (Bio-Rad) and electrophoresed directly on 9% SDS-polyacrylamide gels containing 0.1% gelatin (w/v, from porcine skin, Sigma). Gels were washed 4 times for 20 minutes at room temperature in a 2.5% (v/v) Triton X-100 solution, transferred to a Zymogram development buffer (Bio-Rad) and incubated for 18 hours at 37 °C. Proteins were fixed with 50% methanol/7% acetic acid for 15 minutes and washed (6 times of 5 minutes each) with distilled water. After that, gels were stained for 1 hour with GelCode Blue Stain Reagent (Pierce, Rockford, USA) counterstained with distilled water and then were analyzed with ImageQuant TL (GE Healthcare) after densitometric scanning of the gels. The active MMP-2/(active MMP-2+proMMP-2) intensity ratio designated as the MMP-2 activation ratio. Each zymography assay was repeated at least three times to ensure accuracy.

### Statistical Analysis

Differences in the intravenous treatment groups were assessed with one-way Analysis of Variance (ANOVA) test and independent Student’s t-test with equal variance. Differences between treated and control eyes in the intravitreal treatment groups were assessed with paired Students t-test with equal variance or Wilcoxońs test in cases in which non-parametric tests were required. P<0.05 was considered statistically significant (all statistical analysis performed using SPSS 15.0 software, SPSS Inc., Chicago, IL).

## Results

### Fluorescein Angiography

**Figure 2:**
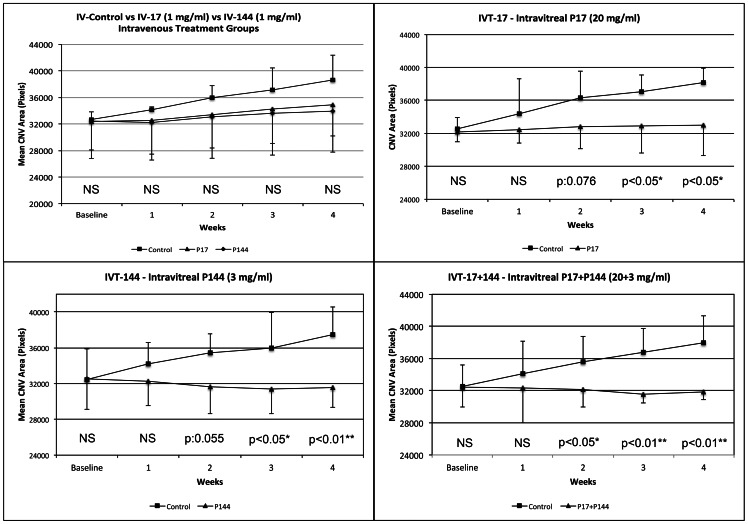
Mean CNV areas assessed by fluorescein angiographies in all study groups (measured in pixels). Differences between treated and untreated eyes. Top left: Intravenous Controls vs Intravenous P17 vs Intravenous P144 (IV-Control vs IV-17 vs IV-144); top right: Intravitreal P17 (IVT-17); bottom left: Intravitreal P144 (IVT-144); bottom right: Intravitreal P17+P144 (IVT-17+144). (NS: Not significant; * p<0.05; ** p<0.01).

The mean CNV area determined for all study groups is summarized in Table 1. In the intravenous treatment groups, no significant differences were found in any of the studied groups. In the intravitreal study groups, mean CNV areas in IVT-17 and IVT-144 groups showed statistically significant differences between controls and treated eyes from the 3^rd^ week. In the IVT-17+144 group, statistically significant differences were found from the 2^nd^ week in treated eyes compared to their respective controls (Figure 2).

**Table 1:** Mean CNV areas assessed by fluorescein angiography (measured in pixels, mean +/− SD).

Study Group		Baseline			1st Week			2nd Week			3rd Week			4th Week		
		Control Eyes	Treated Eyes	p	Control Eyes	Treated Eyes	p	Control Eyes	Treated Eyes	p	Control Eyes	Treated Eyes	p	Control Eyes	Treated Eyes	p
**IV-Control**	**Intravenous Control**	32674 +/−1185	−		34130 +/−4860	−		35972 +/−1770	−		37147 +/−3305	−		38633 +/−3773	−	
**IV-17**	**Intravenous P17**	−	32367 +/−4288	NS	−	32555 +/−5102	NS	−	33441 +/−5117	NS	−	34230 +/−5165	NS	−	34846 +/−4683	NS
**IV-144**	**Intravenous P144**	−	32482 +/−5684		−	32216 +/−5672		−	33023 +/−6194		−	33566 +/−6303		−	33960 +/−6203	
**IVT-17**	**Intravitreal P17**	32551 +/−1407	32170 +/−1190	NS	34387 +/−4273	32438 +/−1614	NS	36300 +/−3235	32807 +/−2671	0.076	37041 +/−2094	32889 +/−3273	<0.05*	38161 +/−1769	33013 +/−3712	<0.05*
**IVT-144**	**Intravitreal P144**	32455 +/−3449	32543 +/−3419	NS	34231 +/−2384	32259 +/−2721	NS	35424 +/−2131	31634 +/−2998	0.055	35964 +/−4012	31398 +/−2783	<0.05*	37473 +/−3060	31540 +/−2212	<0.01**
**IVT-17&144**	**Intravitreal P17&P144**	32498 +/−2691	32391 +/−2464	NS	34143 +/−3971	32325 +/−4323	NS	35642 +/−3101	32116 +/−2180	<0.05*	36754 +/−3002	31560 +/−1086	<0.01**	37929 +/−3436	31887 +/−1004	<0.01**

Mean CNV areas in treated and control eyes at every timepoint for all study groups. Significance level: p<0.05. (NS: Not significant; *:p<0.05; **:p:<0.01).

### Quantitative Real time-Polymerase Chain Reaction (RT-PCR)

**Figure 3:**
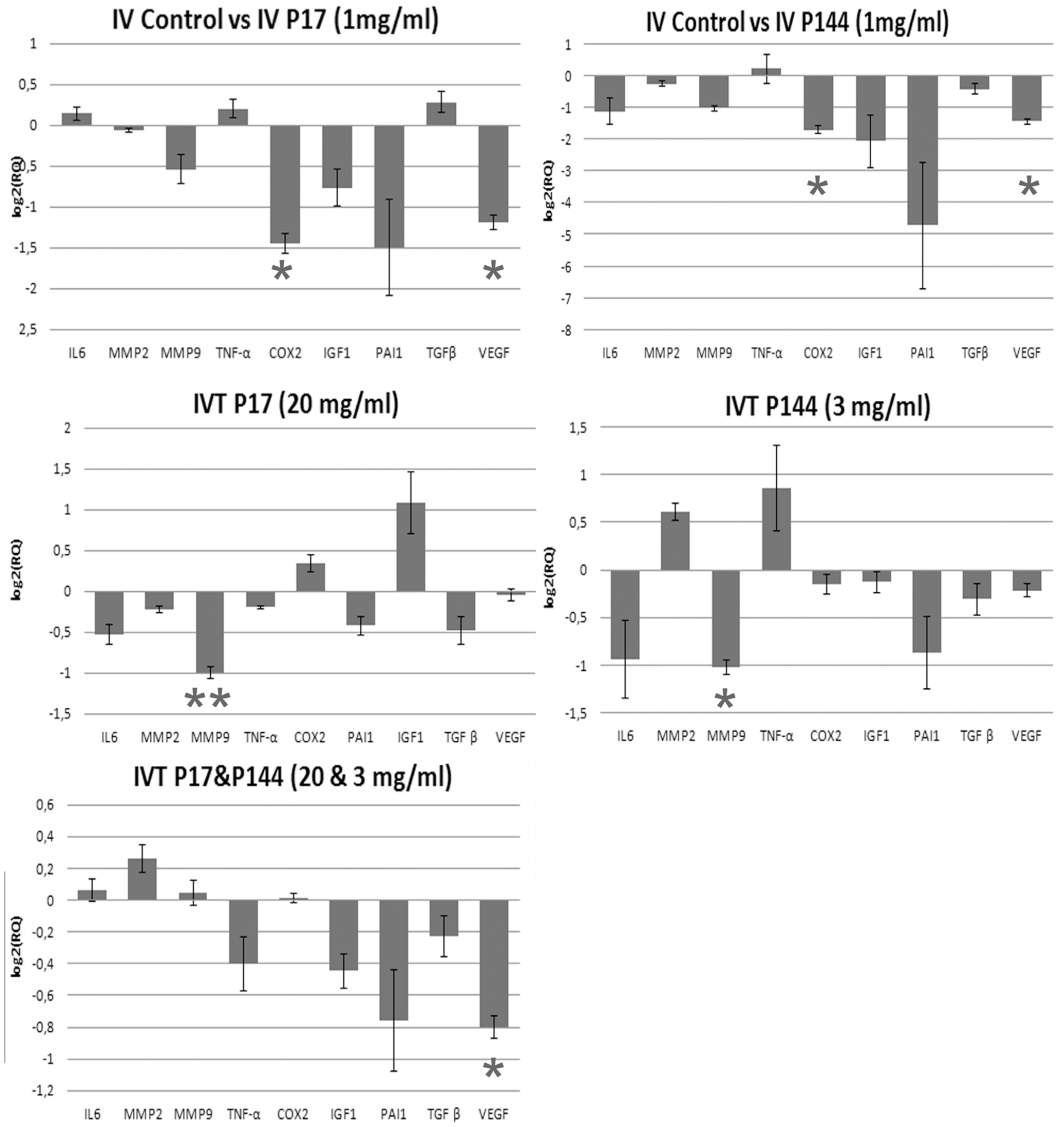
Real time-Polymerase Chain Reaction (Rt-PCR) results. Differences between treated and untreated eyes in all study groups (Mean+/−SD; 5 eyes/study group). Top left: IV-Control vs IV-17. Top right: IV-Control vs IV-144. Middle left: IVT-17. Middle right: IVT-144. Bottom left: IVT-17+144. (NS: Not significant; * p<0.05; ** p<0.01).

Results obtained from all study groups are shown in Figure 3. Significant differences were obtained in VEGF and COX-2 genes in both IV-17 and IV-144 study groups compared to IV-Control group. None of the other studied genes were significantly different in the intravenous study groups. In the intravitreal study groups, significant differences were obtained in MMP-9 gene in treated eyes of IVT-17 and IVT-144 groups compared to their respective controls. No significant differences were obtained in other genes for these groups. In the IVT-17+144 group, differences were significant in VEGF gene between treated and control eyes. All these data are presented in Table 2.

**Table 2:** Real time Polymerase Chain Reaction.

		IV-control vs IV-17		IV-control vs IV-144		IVT-17		IVT-144		IVT-17&144	
Assay	Type	(RQ)	(P-Value)	(RQ)	(P-Value)	(RQ)	(P-Value)	(RQ)	(P-Value)	(RQ)	(P-Value)
b actin	Selected Control	1	NA	1	NA	1	NA	1	NA	1	NA
**IL6**	**Target**	1.102	NS	0.4556	NS	0.6928	NS	0.520	NS	1.043	NS
**MMP-2**	**Target**	0.956	NS	0.8336	NS	0.8545	NS	1.529	NS	1.198	NS
**MMP-9**	**Target**	0.6876	NS	0.4876	NS	0.5007	<0.01**	0.4922	<0.05*	1.030	NS
**TNF-α**	**Target**	1.152	NS	1.152	NS	0.8729	NS	1.806	NS	0.7556	NS
**COX-2**	**Target**	0.3651	<0.05*	0.3051	<0.05*	1.268	NS	0.9001	NS	1.010	NS
**IGF-1**	**Target**	0.5891	NS	0.2391	NS	0.7463	NS	0.9153	NS	0.7339	NS
**PAI-1**	**Target**	0.3559	NS	0.0379	NS	2.114	NS	0.5484	NS	0.5913	NS
**TGF-β**	**Target**	1.213	NS	0.7399	NS	0.7150	NS	0.8074	NS	0.8545	NS
**VEGF**	**Target**	0.4386	<0.05*	0.3636	<0.05*	0.9675	NS	0.861	NS	0.5746	<0.05*

Results are expressed as specific gene Relative Quantity (RQ) and p-value, β-Actin was selected as a control gene (5 eyes/group). Significance level: p<0.05. (NS: Not significant; *:p<0.05 ; **:p:<0.01; NA: Not analyzed).

### Western blotting for vascular endothelial growth factor (VEGF) and phosphorylated SMAD-2

**Figure 4:**
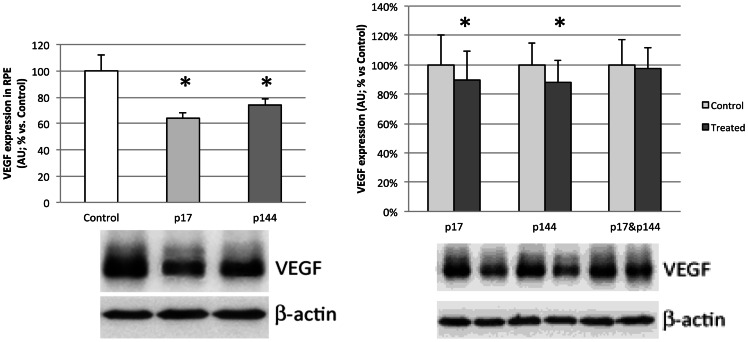
Western blotting for vascular endothelial growth factor (VEGF) expression in RPE in intravenous groups (left) and intravitreal groups (right). Bars represent mean ± SEM of percentage of arbitrary units (AU) vs. control (Total protein loaded: 8 µg; 5–6 eyes/group). (* p<0.05).

**Figure 5:**
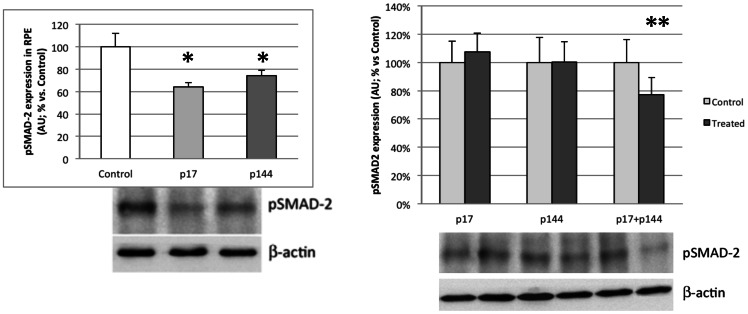
Western blotting for phosphorylated SMAD-2 (pSMAD-2) expression in RPE in intravenous groups (left) and intravitreal groups (right). Bars represent mean ± SEM of percentage of arbitrary units (AU) vs control (Total protein loaded: 8 µg; 5–6 eyes/group).(* p<0.05; ** p<0.01).

Western Blot results for mean VEGF and pSMAD-2 protein levels in treated and control eyes for all study groups are summarized in Tables 3 and 4. Mean VEGF protein expression was significantly lower in the IV-17, IV-144, IVT-17 and IVT-144 treated eyes compared to control eyes (Figure 4). No significant differences were found in the other groups. Mean pSMAD-2 protein levels were significantly lower in the IV-17, IV-144 and IVT-17+144 groups (Figure 5). None of the other study groups showed significant differences between treated and control eyes.

**Table 3:** VEGF protein expression, pSMAD-2 protein levels and MMP-2 activity in RPE homogenates - Intravitreal groups.

	IVT-17		IVT-144		IVT-17+144	
	Control	Treated	Control	Treated	Control	Treated
**VEGF**	100±20	90±20*	100±15	88±14*	100±17	98±14
**pSMAD-2**	100±15	108±13	100±18	100±14	100±16	77±12**
**MMP-2 activity**	100±16	89±12*	100±13	84±12*	100±22	97±18

Data are shown as mean ± SEM of percentage of arbitrary units (AU) vs control eye. (*p<0.05 and **p<0.01).

**Table 4:** VEGF protein expression, pSMAD-2 protein levels and MMP-2 activity in RPE homogenates - Intravenous groups.

	Control	IV-17	IV-144
**VEGF**	100±11	64±6*	74±3*
**pSMAD-2**	100±12	81±4*	87±5*
**MMP-2 activity**	100±5	71±4*	69±4*

Data are shown as mean ± SEM of percentage of arbitrary units (AU) vs control group. (*p<0.05).

### Gelatin zymography assay for matrix metalloproteinase-2 (MMP-2) activity

**Figure 6:**
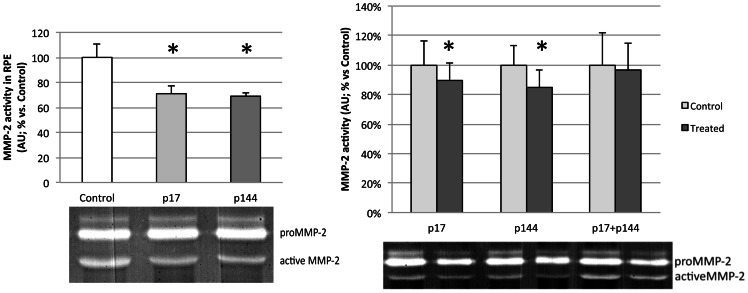
Gelatin zymography assay for matrix metalloproteinase-2 (MMP-2) activity in RPE homogenates from intravenous groups (left) and intravitreal groups (right). Bars represent mean ± SEM of percentage of arbitrary units (AU) vs. control (Total protein loaded: 8 µg; 5–6 eyes/group). (* p<0.05).

MMP-2 activity levels were significantly lower in both intravenous treatment groups IV-17 and IV-144 compared to controls. In the intravitreal study groups, active MMP-2 levels were significantly lower in IVT-17 and IVT-144 study groups (Figure 6). No differences were observed in the IVT-17+144 group. These results are presented in Tables 3 and 4.

## Discussion

The results obtained in our study demonstrate that peptides P17 and P144 reduce early CNV progression in a laser-induced rat model. This is achieved through downregulation of VEGF and TGF-β, but also by affecting other mediators involved in the angiogenic process, such as COX-2, MMP-2 and MMP-9. These findings suggest that TGF-β inhibition may be a novel therapeutic approach, different to that of current treatments for early CNV lesions.

CNV is a complex process that develops in several stages. Different growth factors and cellular types are important in each stage of the pathogenesis of CNV and similarities with wound healing processes have been thoroughly described [10], [33], [34]. The process starts in a favorable environment, commonly with hypoxia, defects in Bruchs membrane, and increased oxidative stress [35]. In these conditions, a pro-inflammatory state is created by the release of interleukins (e.g., IL-6 and IL-8) and important growth factors (e.g., MCP-1 and TGF-β) from RPE cells [36], [37]. These cytokines have a strong chemotactic effect over macrophages, neutrophils and platelets, leading to their infiltration of the area and subsequent enhancement of the early response by acting as a complementary source of growth factors [34], [38]–[41]. During this recruitment, a second stage begins with prominent angiogenesis and fibrotic processes. The main growth factors involved in angiogenesis are VEGF, angiopoietins, MCP-1, b-FGF and TGF-β, which are frequently over-expressed at this stage through complex up-regulation pathways that involve simultaneously several of these mediators, whereas angiogenesis inhibitors such as PEDF or thrombospondin are decreased [10], [16]–[18]. Concomitantly, fibrosis is directed by PDGF, TGF-β, connective tissue growth factor (CTGF) and IGF, leading to incipient tissue regeneration processes in the lesion margins. Finally, the last stage of CNV is the scar tissue formation phase, in which processes of collagen remodelling and scar contraction are mediated by FGF, IGF, CTGF and especially TGF-β [10], [15]–[18]. In this stage, the processes of degradation, synthesis and extracellular matrix remodelling are directed by metalloproteinases such as MMP-2 and MMP-9, and its role in CNV has been described and highlighted by recent studies [42]. The multiple roles played by TGF-β and its relations with other growth factors implicated in the different stages of CNV development led us to evaluate the effect of its inhibition in early CNV lesions [15]–[19].

The results obtained with both peptides in the current study have been promising. Fluorescein angiography analysis showed decreased mean leakage area in all treated groups compared to controls. Although this decrease did not reach the significance level in intravenous groups, differences observed in all intravitreal study groups were significant suggesting an inhibitory effect over fluorescein leakage from laser-induced CNV lesions. Results of biochemical analyses suggested that both peptides behaved in a similar fashion for both intravenous and intravitreal administration. These findings suggest that the pattern of TGF-β inhibition is similar with the two peptides and depends on the administration route, affecting the same mediators in a reproducible way in agreement with previous studies that found similar activity over TGF-β in in-vitro assays [26]. To explain this different behaviour, future pharmakodynamic studies should be directed to assess the duration of peptides at intravitreal or intravenous level. Moreover, the role of the hematoretinal barrier and its effect over the diffusion characteristics of the peptides over the retinal structures has also to be addressed.

The inhibition of VEGF gene expression and decreased VEGF protein expression observed are consistent with our previous study that reported effective downregulation of VEGF with both peptides. These findings confirm that both P17 and P144 peptides may have an antiangiogenic role in CNV treatment affecting the main target of current treatments through TGF-β inhibition. However, two exceptions were observed in our study. In the intravitreal peptide groups no significant differences were found in VEGF gene expression, and intravitreal mixture of both peptides showed differences in VEGF gene expression but not in VEGF protein expression. Whereas the type of study design and more concisely the 6 weeks time from laser induction to eye collection may explain the first, we suggest that joint administration of both peptides may affect its properties and may explain differences compared to single peptide injection. However, it is unclear how this change is produced and again pharmacokinetic studies should be directed to analyse these results. Decreased COX-2 gene expression observed suggests an inhibitory effect of the peptides over the inflammatory process that underlies CNV, and hypothetically may represent an added collateral benefit over single anti-VEGF blocking therapy. Although this finding has not been confirmed in the intravitreal study groups, this potential benefit is biologically plausible regarding the inflammatory conditions of early stages of CNV and must be addressed by future more rigorous studies, including use of specific inhibitors or knock-down of cytokines involved in its signalling pathway. Differences in pSMAD-2 protein levels were obtained in the intravenous treatment groups and combined P17 and P144 intravitreal group. The unstable nature of TGF-β makes its biochemical processing difficult and frequently differences can be overlooked if the primary cytokine is targeted. Therefore, the understanding of its signalling pathway has pointed the pSMAD-2 as a, although still unstable, more reliable marker. Once the TGF-β binds its membrane receptor, a signalling cascade activates the intracellular phosphorylation of SMAD-2 protein, resulting in a pSMAD-2 protein. The measurement of its levels has been accepted as an indirect marker for TGF-β activity. The results obtained in the intravenous groups and intravitreal mixture group confirmed this TGF-β inhibition, although these results were not observed in the single peptide intravitreal groups, probably due to a different pharmacokinetic profile. The effect of the peptides in extracellular matrix remodelling processes was also remarkable. Decreased MMP-9 gene expression was observed in both intravitreal groups and gelatin zymography assays revealed significantly lower MMP-2 activity in the treated eyes of both intravenous and intravitreal groups, suggesting a modulatory effect of both peptides. These findings are especially relevant because extracellular matrix remodelling is prominent from early to late CNV lesions, and may represent an additional benefit compared to current CNV treatments.

A deeper understanding of the different stages of angiogenesis and their respective growth factors and cellular types is warranted for the development of new therapies for CNV. Although intravitreal anti-VEGF results have not been surpassed by any other therapy, due to their multifactorial nature, angiogenesis inhibition alone may not be sufficient to stop the development of CNV lesions [11]–[14]. Thus, new approaches may target mediators involved in CNV at different levels of its etiopathogenesis. Whereas in normal eyes TGF-β is a multifunctional regulator that mediates cellular proliferation, differentiation, apoptotic death and angiogenesis, the multiple roles played in different CNV stages suggest that its blockage not only may decrease the angiogenic process, but also affect other pathways related with the progression of the lesions to scar tissue formation [10], [15]–[18].

The results reported here reinforce the preliminary results obtained in our previous study in CNV induction and raise a significant interest to develop new therapeutic approaches. However, this study has several limitations. Whereas FA is a standardized valid method for CNV assessment, the complementary use of histopathology techniques such as flat mounts may provide an accurate ex-vivo CNV size estimation to support the in vivo findings herein reported. In addition, these techniques may be used to identify and potentially quantify the studied cytokines, allowing an indirect estimation of their downregulation with the peptides. These examinations appear extremely interesting and further studies may be required to confirm or discard the data reported in this work. Interspecies differences between human and rat retina, the unaffected RPE of young healthy rats employed, or interim of time between lesion induction, treatment injection and sample collection appear as other limitations that may have affected the results obtained. However, these features may explain the lack of differences observed in some comparisons, but reinforce the significant results obtained in others. If laser-induced CNV reproduces the stages of the wound-healing process and involves similar growth factors as pathologic CNV, this model may be useful to assess the peptides effect in early CNV lesions, and consequently the inhibition of TGF-β by the peptides should be comparable.

In summary, we report that TGF-β inhibition reduces progression of early CNV lesions in a laser-induced rat model and that this reduction is associated with downregulation of VEGF, TGF-β, COX-2, MMP-2 and MMP-9. The future of CNV management may require combined therapies with multiple drugs acting on different mediators involved in CNV progression, such as VEGF, complement system, TGF-β, PDGF, COX-2, MMPs and TNFα. In this scenario, the anti-TGF-β peptides should be considered in this multivariate CNV treatment, but further studies are needed to assess their effect in established lesions and especially combined with anti-VEGF drugs, the current treatment for this condition.
